# Brain metastasis from gastric adenocarcinoma: A large comprehensive population-based cohort study on risk factors and prognosis

**DOI:** 10.3389/fonc.2022.897681

**Published:** 2022-10-18

**Authors:** Lei Huang, Lei Wang, Yan Shi, Yajie Zhao, Chenying Xu, Jun Zhang, Weiguo Hu

**Affiliations:** ^1^ Department of Oncology, Ruijin Hospital, Shanghai Jiao Tong University School of Medicine, Shanghai, China; ^2^ Medical Center on Aging of Ruijin Hospital (MCARJH), Shanghai Jiao Tong University School of Medicine, Shanghai, China; ^3^ Department of Gastroenterology, Ruijin Hospital, Shanghai Jiaotong University School of Medicine, Shanghai, China; ^4^ Department of Geriatrics, Ruijin Hospital, Shanghai Jiao Tong University School of Medicine, Shanghai, China; ^5^ State Key Laboratory of Oncogenes and Related Genes, Shanghai Jiao Tong University, Shanghai, China

**Keywords:** gastric adenocarcinoma, brain metastasis, survival, cumulative incidence function, Fine-Gray sub-distribution hazard regression, competing risk analysis, large population-based cohort study

## Abstract

**Aims:**

Although brain metastasis from gastric adenocarcinoma (GaC) is rare, it may significantly affect survival and quality of life. The aim of this large, comprehensive, population-based cohort investigation was to investigate factors that were associated with brain metastasis from GaC and to explore the prognostic factors and time-dependent cumulative mortalities among cases with GaC and brain involvement.

**Methods:**

Population-based information on cases with GaC diagnosed from 2010 to 2016 was obtained from a large-scale database. Factors that were associated with brain metastasis were investigated utilizing multivariable logistic regression. Time-dependent tumor-specific mortalities of cases with GaC and brain involvement were then computed utilizing the cumulative incidence functions (CIFs), and mortalities were compared between subgroups utilizing Gray’s test. Factors that were associated with death were further evaluated utilizing multivariable Fine–Gray subdistribution hazard regression.

**Results:**

Together, 28,736 eligible cases were included, which comprised 231 (1%) cases with brain metastasis and 10,801 (38%) with metastasis to other sites, encompassing a follow-up of 39,168 person-years. Brain metastasis occurred more often among younger patients (within overall cancers), in cases with stomach cardia tumors, within cases with signet-ring cell carcinoma (within overall cancers), and within cases with positive lymph nodes (within overall tumors); it was less often detected among black people. Brain involvement was associated with more lung and bone metastases. The median survival time of cases having brain metastasis was only 3 months; the 6- and 12-month tumor-specific cumulative mortalities were 57% and 71%, respectively. Among cases with GaC and brain metastasis, those with gastric cardia cancers (when receiving radiotherapy), those undergoing resection, and those receiving chemotherapy had lower mortality risks, while younger patients (when receiving chemotherapy or radiotherapy) and people with positive lymph nodes (when receiving radiotherapy) had higher death hazards.

**Conclusion:**

Among patients with GaC, brain metastasis was correlated with several clinical and pathological variables, including ethnicity, age, cancer histology, location, lymph node involvement, and metastases to other sites. Cases having brain metastasis had poor survival that was correlated with age, cancer location, lymph node metastasis, and management. These findings offer vital clues for individualized patient care and future mechanistic explorations.

## Background

Gastric cancer, most of which is gastric adenocarcinoma (GaC), is the fifth most common malignancy and the fourth most frequent cause of cancer-associated death worldwide, with approximately 1,100,000 new incident cases and about 800,000 related mortalities estimated in 2020 (1–4). Metastatic GaCs (mGaCs) with malignant involvement of distant sites take up about 1/3 of all GaCs on diagnosis and are associated with inferior survival because of aggressive progression (1, 5–7).

We previously reported that within cases with mGaC, the proportions of people having brain metastasis (BM) were 2% and 1% in the USA and the Netherlands, respectively, among overall patients with mGaC, and 1% and <1%, respectively, within those with resected mGaC (5). Although BM is rare among mGaCs, it may have been underestimated (8–11). BM from GaC was first reported in 1960 (12) and can occur synchronously with, or early, or even years after, curative resection for early GaCs because of residual micrometastases, and its incidence keeps increasing as disease-associated prognosis improves with the advancement of treatment (13–18). BM represents a severe condition and may cause early onset of neurological symptoms, markedly undermining prognosis, and deteriorating quality of life (QoL) (19–22), and it can even cause the initial symptoms of gastric cancer (23–26). The prognosis of patients with GaC involving the central nervous system remains very poor (27–29), and early identification of BM is vital for guiding clinical treatment and preventing related complications. People with GaC and BM represent a unique and largely heterogeneous population (30–33), and there have been few studies with a sufficient number of cases pertaining to the factors that were associated with BM and its prognostic significance within cases with GaC, which might be because of the underestimation of diagnosis and/or rarity of the disease.

In this comprehensive investigation, a large-scale population-based cohort was analyzed to delineate features of GaC cases having BM, who were compared with cases having distant metastasis that spared the brain and those without distant metastasis, and to reveal factors that were associated with BM in different subgroups of GaC cases. We further computed GaC-specific cumulative mortalities at various follow-up time-points with the cumulative incidence function (CIF) and explored prognostic factors with competing risk (CR) regression among patients with GaC and BM. The findings might help with individualized care for this special patient group.

## Methods

### Participants

After the corresponding agreement was signed and the data utilization permission was obtained, individual case-level data were obtained from the Surveillance, Epidemiology, and End Results-18 database of the National Cancer Institute, which collects data from several population-based cancer registries and is an authoritative source of data pertaining to the US cancer statistics. The National Cancer Institute staff works together with the North American Association of Central Cancer Registries to guide all of the state registries to provide compatible data allowable for pooling. The database is the only comprehensive source of US population-based cancer data that includes tumor stage on diagnosis and individual survival information (34).

Patients with pathologically verified primary invasive stomach adenocarcinoma diagnosed in 2010–2016 were included (**Supplementary Figure S1**). We determined the enrollment time period by considering the availability of information on the metastatic site. We included signet-ring cell carcinoma (SRC), which is a special type of adenocarcinoma (3). We excluded patients with prior cancers, with a diagnosis based on autopsy or death certificate only, and/or with ineligible histology (neuroendocrine tumor/carcinoid, gastrointestinal stromal tumor (GIST)/sarcoma, squamous cell cancer, lymphoma, and germ-cell neoplasm; **Supplementary Table S1**). To enable analyses of CRs, cases with unclear causes of mortality were excluded. We excluded patients with unclear BM or distant involvement status, considering that they did not contribute to addressing the study focus-BM.

The investigated registry routinely registers information pertaining to case demographics (e.g., ethnicity, age, sex, and year of diagnosis), primary cancer location, stage on diagnosis, cancer morphology (from which differentiation and histology could be derived), first-course management (resection, chemotherapy, and radiation therapy), cause of mortality, and survival status and time, which were included for analyses in this investigation. The National Center for Health Statistics provided the survival information. Nonresection treatment information was under-ascertained with low sensitivity (35). We dichotomized the year of diagnosis into two time periods: 2014–2016 and 2010–2013, and categorized age into five subgroups: ≥80, 70–79, 60–69, 50–59, and <50 years. Cancer location contained four categories: gastric antrum/pylorus, fundus/body, cardio, and others.

### Analyses

Continuous variables were presented as mean ± standard deviation, median (interquartile range), and categorical data were shown as count [percentage (%)]; the patient, cancer, management, and prognosis features for cases with GaC and BM, cases with distant involvement that spared the brain, and cases without distant involvement were described. We calculated survival time from initial diagnosis until mortality or last follow-up, whichever took place first, and estimated follow-up time utilizing the reverse Kaplan–Meier method (36).

We quantified the correlations of BM versus no brain and/or distant involvement with age, sex, ethnicity, year of diagnosis, cancer location, SRC histology, and liver, lung, and bone involvements with the multivariable logistic regression, mutually adjusting for these factors, among overall and metastatic tumors. For cancer differentiation and adjacent structure and lymph node (LN) involvements with missing data, we assessed the correlations *via* additionally adding these factors one by one into the abovementioned model.

Mortalities were calculated with the CIFs (37, 38), where, different from the routine Kaplan–Meier method, estimation of an event incidence for cancer-specific mortalities while taking into account CRs from other causes of mortality is allowable. We computed cumulative mortalities overall and categorized them by patient, cancer, and management factors at follow-ups of 6 and 12 months, and assessed differences in mortality between groups with Grays’ test for CIF equality.

Considering that incidence of death was focused on in this study rather than etiology, Fine–Gray subdistribution hazard regression (37, 38) was applied to investigate prognostic factors and to compare the cumulative hazards of tumor-related mortalities across subgroups with different individual prognostic and risk factors within overall GaC cases with BM, and the corresponding hazard ratios (HR_SD_) and 95% confidence intervals (CIs) were computed; we mutually adjusted the model for ethnicity, age group, sex, period of diagnosis, cancer location, SRC histology, liver, lung, and bone involvement, and surgical resection. For cancer differentiation, and adjacent structure and LN involvements with missing data, we assessed the correlations by additionally adding these factors one by one into the abovementioned model. We did not incorporate nonsurgical variables into the multivariable-adjusted models due to the low sensitivity and under-ascertainment (35) and did stratified analyses for cases undergoing chemotherapy or radiation therapy. Before doing modeling survival analyses, we confirmed the proportional hazard (PH) assumption both analytically utilizing the scaled Schoenfeld residuals test and graphically utilizing the log–log plot (39). The univariable survival curves for both overall cases categorized by the brain and distant involvement and cases having BM categorized by patient and cancer factors were plotted. We did analyses utilizing the R 4.1.2 software (http://cran.r-project.org) and considered the findings to be statistically significant with a two-sided *p*-value <0.05.

## Results

### Features of participants

**Figure 1:**
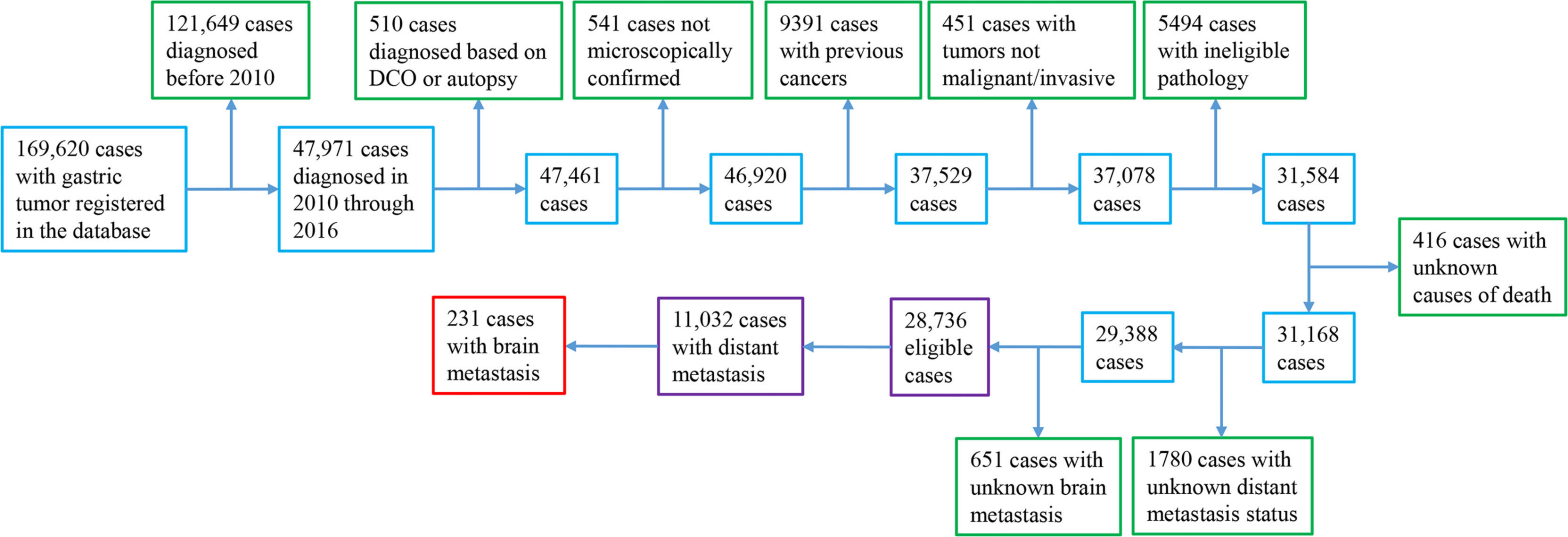
Case selection flow diagram.

Within 169,620 registered patients with gastric cancer, 11,032 cases with mGaC and clear BM status and 17,704 with nonmetastatic GaC who were diagnosed in 2010 through 2016 were eligible, together encompassing a follow-up of 39,168 person-years; within patients with metastatic disease, 231 (2%) had BM (**Figure 1**). When compared with cases having metastasis that spared the brain and those with nonmetastatic cancer, patients having BM were slightly more frequently diagnosed in 2014 or later (46% versus 45% and 43%), of younger age (mean age, 61 versus 63 and 68 years), and of male gender (73% versus 64% and 64%) (**Table 1**). The proportion of cases ≥80 years was smaller (8% versus 14% and 22%) in people with BM, while the proportion of cases <50 years was greater (19% versus 16% and 9%). Cases having BM were more frequently of white ethnicity (82% versus 71% and 69%) and had more frequently gastric cardia tumors (75% versus 52% and 49%) but less frequent gastric antrum/pylorus tumors (8% versus 27% and 34%). Tumors with BM often had more positive LNs (60% versus 57% and 47%). Within metastatic cancers, those with BM also more often had metastases to the lung (33% versus 14%) and bone (32% versus 13%), but less frequently to the liver (36% versus 43%). Resection was less frequently done for cases with BM (7% versus 11% and 64%). The median survival time was 3, 5, and 25 months for cases with BM, cases with nonbrain metastasis, and cases without distant metastasis, respectively, and 13%, 16%, and 48% of cases, respectively, survived in the three groups at the cutoff of follow-up (**Supplementary Figure S1**).

**Table 1:** Baseline features of cases with metastatic (with and without brain involvement) and nonmetastatic gastric adenocarcinoma, 2010 through 2016.

Variable	Category/comment	Metastatic		Nonmetastatic
		With brain metastasis	Without brain metastasis	
***n***		231	10,801	17,704
**Year of diagnosis**	2014–2016	107 (46)	4,888 (45)	7,582 (43)
**Sex**	Male	168 (73)	6,943 (64)	11,276 (64)
**Age** (years)	As continuous	61 ± 14, 61 (53–70)	63 ± 14, 64 (54-74)	68 ± 14, 69 (59-78)
	<50	44 (19)	1,742 (16)	1,647 (9)
	50–59	58 (25)	2,402 (22)	2,983 (17)
	60–69	68 (29)	2,939 (27)	4,529 (26)
	70–79	42 (18)	2,233 (21)	4,566 (26)
	≥80	19 (8)	1,485 (14)	3,979 (22)
**Ethnicity**	White	190 (82)	7,721 (71)	12,222 (69)
	Black	16 (7)	1,463 (14)	2,234 (13)
	American Indian/Alaska Native	2 (1)	127 (1)	168 (1)
	Asian/Pacific Islander	23 (10)	1,435 (13)	2,963 (17)
	Other unspecified/unknown	0 (0)	55 (1)	117 (1)
**Tumor location** ^a^	Gastric cardia	120 (75)	3,562 (52)	6,227 (49)
	Gastric fundus/body	26 (16)	1,403 (21)	2,104 (17)
	Gastric antrum/pylorus	13 (8)	1,820 (27)	4,342 (34)
	Other	72 (31)	4,016 (37)	5,031 (28)
**Signet ring cell carcinoma**	Yes	46 (20)	2,417 (22)	3,223 (18)
**Differentiation** ^b^	Well	3 (2)	165 (2)	940 (6)
	Intermediate	56 (34)	1,839 (22)	4,448 (29)
	Poor/undifferentiated	107 (64)	6,406 (76)	9,977 (65)
**Adjacent structure invasion** ^c^	Yes	23 (23)	1,575 (26)	1,174 (7)
**Positive lymph node** ^d^	Yes	108 (60)	5,110 (57)	8,036 (47)
**Bone metastasis**	Yes	74 (32)	1,377 (13)	0 (0)
**Liver metastasis**	Yes	84 (36)	4,611 (43)	0 (0)
**Lung metastasis**	Yes	76 (33)	1,541 (14)	0 (0)
**Resection**	Yes	16 (7)	1,160 (11)	11,301 (64)
**Chemotherapy** ^e^	Yes	138 (60)	6,530 (60)	9,119 (52)
**Radiotherapy** ^e^	Yes	142 (61)	1,704 (16)	5,912 (33)
**Follow-up** (months)^f^	As continuous	33 (17–49)	36 (16–59)	39 (18–60)
**Accumulated follow-up** (person-years)	As continuous	110	6,867	32,191
**Median survival** (months)	As continuous	3 (1–8)	5 (1–12)	25 (8–NE)
**Cause of death**	Alive	31 (13)	1,678 (16)	8,484 (48)
	Cancers	193 (84)	8,739 (81)	7,786 (44)
	Noncancer diseases	7 (3)	384 (4)	1,434 (8)

Categorical data are shown as count [percentage (%)], while continuous data are shown as mean ± standard deviation and median (interquartile range). Records are complete, unless otherwise specified below.

a The proportions of cancers located in gastric cardia, fundus/body, and antrum/pylorus were calculated within these three categories. Other means lesser curvature, greater curvature, overlapping lesion of the stomach, and stomach (not otherwise specified).

b Missing differentiation grade: metastatic with brain metastasis, 65 (28%); metastatic without brain metastasis, 2,391 (22%); nonmetastatic, 2,339 (13%).

c Missing local invasion: metastatic with brain metastasis, 133 (58%); metastatic without brain metastasis, 4,714 (44%); nonmetastatic, 2,018 (11%).

d Missing positive lymph node: metastatic with brain metastasis, 51 (22%); metastatic without brain metastasis, 1,797 (17%); nonmetastatic, 564 (3%).

e The other category for the nonsurgical variables was “No/unknown,” considering the low sensitivity.

f Shown as median (interquartile range) and computed utilizing the reverse Kaplan–Meier method.

NE, not estimable.

### Brain metastasis-associated factors

Among cases with mGaC (**Table 2**), BM less often occurred in black people (OR = 0.57) and those with gastric antrum/pylorus tumors (OR = 0.28); it was more often correlated with bone (OR = 2.60) and lung involvements (OR = 2.56), but less frequently liver involvement (OR = 0.64). Within overall cancers, the patterns and strengths of associations were mostly similar with those in metastatic diseases, with some exceptions: The trend of the negative association between age and BM became significant, and octogenarians significantly had often less BM compared to patients aged 60–69 years (versus other nonbrain or no metastasis, OR = 0.55; versus no metastasis, OR = 0.33). The association with black ethnicity became insignificant when versus no metastasis. Neither SRC carcinoma nor LN metastasis was not significantly correlated with BM among metastatic tumors, while they became significantly associated with an increased risk of BM among overall cancers when versus no metastasis (OR = 1.94 and 1.69, respectively). When versus other nonbrain or no metastasis, the strength of the association with BM became stronger (OR = 4.87); BM also turned out to be positively associated with liver metastasis (OR = 1.44).

**Table 2:** Factors associated with brain metastasis versus no brain metastasis (nonbrain or no metastasis) in cases with metastatic or overall gastric adenocarcinoma, 2010–2016.

		Within metastatic cancers			Within overall cancers					
Variable	Category	Versus other nonbrain metastasis			Versus other nonbrain or no metastasis			Versus no metastasis		
		OR (95% CI)	*p*	*p*_trend_	OR (95% CI)	*p*	*p*_trend_	OR (95% CI)	*p*	*p*_trend_
**Year of diagnosis**	2010–2013	1.00 (reference)			1.00 (reference)			1.00 (reference)		
	2014–2016	1.00 (0.77–1.30)	0.998		1.02 (0.78–1.33)	0.896		1.08 (0.71–1.64)	0.729	
**Sex**	Male	1.00 (reference)			1.00 (reference)			1.00 (reference)		
	Female	0.80 (0.59–1.08)	0.150		0.84 (0.62–1.14)	0.250		0.88 (0.54–1.43)	0.603	
**Age** (years)	<50	1.22 (0.82–1.81)	0.321	0.227	1.41 (0.95–2.10)	0.086	**0.004**	1.41 (0.75–2.66)	0.286	**0.003**
	50–59	1.11 (0.77–1.58)	0.584		1.20 (0.84–1.72)	0.323		1.16 (0.67–2.00)	0.605	
	60–69	1.00 (reference)			1.00 (reference)			1.00 (reference)		
	70–79	0.88 (0.59–1.30)	0.505		0.78 (0.53–1.16)	0.222		0.50 (0.27–0.95)	**0.035**	
	≥80	0.67 (0.40–1.13)	0.136		0.55 (0.33–0.92)	**0.023**		0.33 (0.14–0.76)	**0.009**	
**Ethnicity**	White	1.00 (reference)		0.269	1.00 (reference)		0.177	1.00 (reference)		0.851
	Black	0.57 (0.34–0.97)	**0.037**		0.56 (0.33–0.94)	**0.029**		0.62 (0.26–1.44)	0.264	
	American Indian/Alaska Native	0.62 (0.15–2.55)	0.503		0.60 (0.14–2.47)	0.474		–^a^	–^a^	
	Asian/Pacific Islander	0.81 (0.52–1.27)	0.352		0.74 (0.47–1.16)	0.185		0.89 (0.46–1.72)	0.730	
**Tumor location**	Gastric cardia	1.00 (reference)		**<0.001**	1.00 (reference)		**<0.001**	1.00 (reference)		**0.001**
	Gastric fundus/body	0.66 (0.42–1.03)	0.067		0.77 (0.49–1.20)	0.248		0.61 (0.30–1.25)	0.179	
	Gastric antrum/pylorus	0.28 (0.16–0.51)	**<0.001**		0.28 (0.16–0.51)	**<0.001**		0.10 (0.03–0.31)	**<0.001**	
	Other^b^	0.62 (0.45–0.85)	**0.003**		0.77 (0.56–1.06)	0.103		0.66 (0.39–1.10)	0.113	
**Signet ring cell carcinoma**	No	1.00 (reference)			1.00 (reference)			1.00 (reference)		
	Yes	0.85 (0.60–1.20)	0.353		1.06 (0.75–1.50)	0.759		1.94 (1.20–3.15)	**0.007**	
**Differentiation**	Well	1.09 (0.33–3.53)	0.892	**0.003**	0.57 (0.18–1.82)	0.341	0.052	0.28 (0.04–2.07)	0.213	0.189
	Intermediate	1.85 (1.30–2.65)	**0.001**		1.46 (1.02–2.08)	**0.037**		1.39 (0.80–2.42)	0.248	
	Poor/undifferentiated	1.00 (reference)			1.00 (reference)			1.00 (reference)		
**Adjacent structure invasion**	No	1.00 (reference)			1.00 (reference)			1.00 (reference)		
	Yes	1.01 (0.63–1.64)	0.954		1.42 (0.86–2.35)	0.166		1.22 (0.37–4.03)	0.749	
**Positive lymph node**	No	1.00 (reference)			1.00 (reference)			1.00 (reference)		
	Yes	0.94 (0.69–1.29)	0.712		1.03 (0.76–1.41)	0.832		1.69 (1.00–2.85)	**0.049**	
**Bone metastasis**	No	1.00 (reference)			1.00 (reference)			–^a^		
	Yes	2.60 (1.95–3.47)	**<0.001**		4.87 (3.58–6.63)	**<0.001**		–^a^	–^a^	
**Liver metastasis**	No	1.00 (reference)			1.00 (reference)			–^a^		
	Yes	0.64 (0.48–0.86)	**0.003**		1.44 (1.06–1.95)	**0.021**		–^a^	–^a^	
**Lung metastasis**	No	1.00 (reference)			1.00 (reference)			–^a^		
	Yes	2.56 (1.92–3.41)	**<0.001**		4.38 (3.19–6.03)	**<0.001**		–^a^	–^a^	

Odds ratios and 95% confidence intervals for the variables listed in the first column except tumor differentiation were calculated utilizing the logistic regression model mutually adjusted for these variables. For tumor differentiation, adjacent structure invasion, and positive lymph nodes with missing values, the association was assessed by additionally including these variables one by one into the above multivariable-adjusted model. Statistically significant p-values are highlighted in bold.

a Not estimable.

b Lesser curvature, greater curvature, overlapping lesion of the stomach, and stomach (not otherwise specified).

OR, odds ratio; CI, confidence interval.

### Overall and stratified survival of cases with GaC and brain involvement

**Figure 2:**
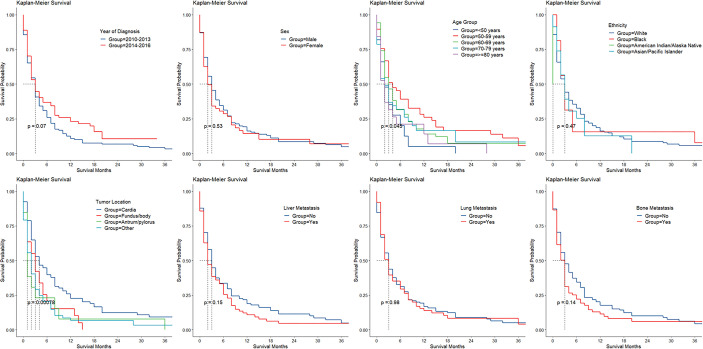
Kaplan–Meier survival by patient and cancer factors in gastric adenocarcinoma with brain involvement.

The Kaplan–Meier survival of cases with BM stratified by age group, sex, ethnicity, period of diagnosis, cancer location, and liver, lung, and bone involvement is shown in **Figure 2**. Utilizing CIFs, overall and categorized tumor-specific mortalities were computed for cases with GaC and BM (**Table 3**). The speed of increment in death slowed down with a longer follow-up period. Within overall cases, the 6-month death rate already was as high as 57%, and the 12-month death rate was 71%. Death rates categorized by ethnicity, age group, sex, cancer location, SRC histology, differentiation grade, adjacent structure invasion, LN, bone, liver, and lung involvement, resection, or radiotherapy were not significantly different across subgroups, while cases receiving chemotherapy had lower death rates than their counterparts within the first year of follow-up (6 months, 45% versus 76%; 12 months, 65% versus 79%).

**Table 3:** Cumulative incidences of cancer-specific mortality (%) at various follow-up points in cases with gastric adenocarcinoma and brain metastasis, overall and categorized.

		Follow-up time		*p* ^a^
Group	Category	6 months	1 year	
		CIM (95% CI)	CIM (95% CI)	
**Overall**		57 (51–64)	71 (64–76)	
**Sex**	Male	56 (48–64)	70 (62–77)	0.695
	Female	60 (46–71)	72 (58–82)	
**Age** (years)	<50	68 (51–80)	83 (67–92)	0.055
	50–59	52 (38–64)	63 (48–75)	
	60–69	58 (44–69)	73 (60–83)	
	70–79	49 (33–63)	61 (43–75)	
	≥80	68 (42–85)	75 (45–90)	
**Ethnicity**	White	55 (48–62)	70 (63–76)	0.247
	Asian/Pacific Islander	62 (38–79)	74 (47–89)	
**Tumor location**	Gastric cardia	52 (43–61)	68 (58–76)	0.382
	Gastric fundus/body	72 (47–86)	77 (51–90)	
	Gastric antrum/pylorus	62 (29–82)	77 (39–93)	
	Other^a^	60 (48–70)	70 (58–80)	
**Signet ring cell carcinoma**	No	56 (48–63)	69 (61–75)	0.256
	Yes	63 (47–75)	78 (61–88)	
**Differentiation**	Intermediate	52 (38–65)	64 (48–75)	0.052
	Poor/undifferentiated	64 (54–73)	76 (66–84)	
**Adjacent structure invasion**	No	52 (40–63)	68 (56–78)	0.635
	Yes	62 (38–79)	77 (37–93)	
**Positive lymph node**	No	47 (35–59)	67 (54–77)	0.205
	Yes	58 (48–67)	69 (59–77)	
**Bone metastasis**	No	54 (46–62)	69 (60–76)	0.406
	Yes	64 (52–74)	75 (63–83)	
**Liver metastasis**	No	55 (46–63)	68 (59–75)	0.424
	Yes	61 (50–71)	74 (63–83)	
**Lung metastasis**	No	57 (49–64)	70 (61–77)	0.997
	Yes	58 (46–69)	72 (60–81)	
**Resection**	No	59 (52–65)	72 (66–78)	0.057
	Yes	33 (11–57)	47 (20–70)	
**Chemotherapy**	No/unknown	76 (66–83)	79 (69–86)	**0.014**
	Yes	45 (36–53)	65 (56–73)	
**Radiotherapy**	No/unknown	62 (51–71)	76 (65–84)	0.294
	Yes	54 (45–62)	67 (58–75)	

Calculated utilizing the cumulative incidence function. Statistically significant p-values are highlighted in bold.

a Utilizing Gray’s test for equality of cumulative incidence functions.

CIM, cumulative incidence of mortality; CI, confidence interval.

### Prognostic factors in GaC cases with brain involvement

Utilizing the Fine–Gray subdistribution hazard function models, factors that were associated with tumor-specific death in GaC patients having BM are listed in **Table 4**. In overall patients, those who underwent resection had a 52% unit reduction in death risk. In subgroups of patients receiving chemotherapy, septuagenarians had a significantly lower mortality risk compared to patients aged 60–69 years (HR_SD_ = 0.39). Among patients receiving radiotherapy, those <50 years had a significantly higher mortality risk compared to those aged 60–69 years (HR_SD_ = 2.03), and individuals with gastric antrum/pylorus tumors had significantly a higher death hazard compared with those with gastric cardia tumors (HR_SD_ = 4.63). LN involvement was associated with a 0.77-unit increase in death risk.

**Table 4:** Fine–Gray subdistribution hazard ratios for cancer-specific mortality among cases with gastric adenocarcinoma and brain metastasis in overall patients and those treated with chemotherapy or radiotherapy.

Variable	Category	Overall			Chemotherapy			Radiotherapy		
		HR (95% CI)	*p*	*p*_trend_	HR (95% CI)	*p*	*p*_trend_	HR (95% CI)	*p*	*p*_trend_
**Period of diagnosis**	2010–2013	1.00 (reference)			1.00 (reference)			1.00 (reference)		
	2014–2016	0.77 (0.58–1.03)	0.080		0.69 (0.46–1.04)	0.076		0.83 (0.55–1.24)	0.360	
**Sex**	Male	1.00 (reference)			1.00 (reference)			1.00 (reference)		
	Female	0.96 (0.67–1.37)	0.803		0.93 (0.60–1.44)	0.750		1.04 (0.63–1.69)	0.891	
**Age group**	<50 years	1.29 (0.85–1.96)	0.237	0.192	1.18 (0.64–2.18)	0.598	0.072	2.03 (1.14–3.62)	**0.016**	**0.039**
	50–59 years	0.87 (0.60–1.24)	0.428		0.92 (0.55–1.55)	0.765		1.02 (0.60–1.73)	0.943	
	60–69 years	1.00 (reference)			1.00 (reference)			1.00 (reference)		
	70–79 years	0.75 (0.45–1.24)	0.264		0.39 (0.18–0.83)	**0.015**		1.48 (0.78–2.80)	0.226	
	≥80 years	1.37 (0.79–2.38)	0.268		0.84 (0.37–1.92)	0.685		2.07 (0.85–5.03)	0.109	
**Tumor location**	Gastric cardia	1.00 (reference)		0.489	1.00 (reference)		0.630	1.00 (reference)		**<0.001**
	Gastric fundus/body	1.28 (0.85–1.93)	0.231		1.27 (0.71–2.26)	0.426		1.48 (0.81–2.69)	0.204	
	Gastric antrum/pylorus	1.17 (0.56–2.42)	0.680		0.81 (0.28–2.36)	0.692		4.63 (2.70–7.96)	**<0.001**	
	Other^a^	0.91 (0.61–1.31)	0.604		0.88 (0.51–1.51)	0.643		1.04 (0.62–1.77)	0.878	
**Signet ring cell carcinoma**	No	1.00 (reference)			1.00 (reference)			1.00 (reference)		
	Yes	1.23 (0.80–1.89)	0.355		1.60 (0.92–2.79)	0.097		0.98 (0.52–1.85)	0.944	
**Differentiation**	Intermediate	0.89 (0.59–1.35)	0.585		0.67 (0.35–1.26)	0.611		0.99 (0.54–1.82)	0.962	
	Poor/undifferentiated	1.00 (reference)			1.00 (reference)			1.00 (reference)		
**Adjacent structure invasion**	No	1.00 (reference)			1.00 (reference)			1.00 (reference)		
	Yes	0.70 (0.35–1.40)	0.309		0.68 (0.22–2.12)	0.503		0.40 (0.11–1.47)	0.167	
**Positive lymph node**	No	1.00 (reference)			1.00 (reference)			1.00 (reference)		
	Yes	1.38 (0.95–1.99)	0.088		1.27 (0.76–2.10)	0.366		1.77 (1.07–2.93)	**0.027**	
**Bone metastasis**	No	1.00 (reference)			1.00 (reference)			1.00 (reference)		
	Yes	1.12 (0.82–1.53)	0.473		1.53 (0.98–2.38)	0.062		1.06 (0.68–1.65)	0.786	
**Liver metastasis**	No	1.00 (reference)			1.00 (reference)			1.00 (reference)		
	Yes	1.25 (0.88–1.76)	0.217		1.55 (0.91–2.65)	0.108		1.15 (0.71–1.85)	0.572	
**Lung metastasis**	No	1.00 (reference)			1.00 (reference)			1.00 (reference)		
	Yes	1.01 (0.73–1.40)	0.941		1.19 (0.76–1.86)	0.456		1.07 (0.69–1.66)	0.765	
**Resection**	No	1.00 (reference)			1.00 (reference)			1.00 (reference)		
	Yes	0.48 (0.26–0.88)	**0.018**		0.35 (0.18–0.68)	**0.002**		0.41 (0.19–0.89)	**0.023**	

Hazard ratios and 95% confidence intervals for the variables listed in the first column except tumor differentiation were calculated utilizing the Fine–Gray subdistribution hazard model mutually adjusted for these variables. For tumor differentiation, adjacent structure invasion, and positive lymph nodes with missing values, the association was assessed by additionally including these variables one by one into the above multivariable-adjusted model. Statistically significant p-values are highlighted in bold.

a Lesser curvature, greater curvature, overlapping lesion of the stomach, and stomach (not otherwise specified).

HR, hazard ratio; CI, confidence interval; NE, not estimable due to small case number.

## Discussion

In our large population-based cohort investigation, 231 patients with BM were identified within about 30,000 cases with GaC encompassing a follow-up of about 40,000 person-years, and their characteristics were described comprehensively, with comparison to those with distant involvement sparing brain or without distant metastasis. Unique risk characteristics specific to BM were identified and further verified with multivariable-adjusted analyses. Taking into account CRs, the overall and categorized tumor-specific death rates of cases with GaC and BM were further shown utilizing CIFs, representing a more trustable prognosis estimation. We further supported comparisons of cumulative death rates by utilizing multivariable-adjusted CR analyses. The findings provide vital hints for screening and treatment of BM from GaCs.

Patients having BM comprised 1% and 2% of cases with overall and metastatic GaC, respectively. While much rarer than metastases to other sites like the liver and lung and from other malignancies like melanoma, lung, and breast cancers, BM from GaC is quickly progressing and could markedly deteriorate survival and undermine QoL (40–42). The overall cumulative death rate increased quickly to 57% at 6 months and 71% at 12 months, with a median overall survival of merely 3 months. With the advancements in techniques of diagnosis and treatment, BM was more often detected and had an enhanced prognosis in more recent years. Long-term survival beyond 5 years may be possible in a small minority of selected brain-metastatic GI cancer patients with specific characteristics, including only one brain metastasis without metastasis to extracranial sides, age <60 years, Karnofsky performance status score of 100, surgical resection, brain metastasis-directed treatment, and additional systemic therapy (43).

There are some methods to identify BM from GaC. BM arising from gastric cancer is frequently revealed by hypointensity on T2-weighted MRI, which may be due to the accumulation of collagen in the tissues (44). FDG-PET can provide additional information on BM (45). Measurements of pre- and postoperative brain blood flow may be helpful in the case of chronic subdural hematoma after dural metastasis from gastric cancer (46). Cerebrospinal fluid cytology may also be used to diagnose leptomeningeal metastasis from HER2+ gastric cancer (47). The peroxidase-antiperoxidase method for carcinoembryonic antigen (CEA) may increase the diagnostic accuracy of cytology in the cerebrospinal fluid for BM from GaC (48). Despite these methods, it remains challenging to detect BM from GaC early, which could often be underestimated and/or misdiagnosed. There may be a great intrapatient heterogeneity in circulating cancer cells at the single-cell level in the cerebrospinal fluid of patients with gastric cancer and BM (49). The expressions of ZEB2 and miRNA-200 family members are correlated with BM from GaC (50). Nonetheless, the driving force behind BM remains largely unclear. It would be important to investigate risk factors associated with BM to facilitate early and efficient detection of this clinically significant but rare condition (51).

We found that BM from GaC occurred more often with younger age, nonblack ethnicities, gastric cardiac location, SRC histology, and LN involvement. Younger patients, cases having antrum/pylorus tumors, and cases having LN involvement had increased death hazards, while surgical resection was associated with a lower mortality hazard. Of note, cases younger than 50 years comprised 19% of all cases with GaC and BM compared with 8% for cases who were ≥80 years. Older cases had generally slower tumor progression, and the preference of BM for younger cases with longer anticipated survival is worthy of doctors’ attention. Cardiac tumors, which are different from noncardiac cancers in various aspects, are correlated with poorer prognosis (18), and we found them to be more frequently correlated with BM, which was supported by an institution-based study (52). Interestingly, they were correlated with higher survival rates among cases with GaC and BM who received radiotherapy. SRC, which is a special histology type, might have higher biologic aggressiveness and lower sensitivity to systemic therapy, making immediate upfront resectional surgery of critical value. While positive LN was more often correlated with BM, 40% of patients with GaC and BM had uninvolved LNs. LN involvement rather than adjacent structure involvement was associated with prognosis among patients with GaC and BM receiving radiotherapy. GaCs with multiple metastasis sites including the brain are noteworthy clinically, while bone, liver, or lung involvement was not correlated significantly with worse survival among patients with GaC and BM in this investigation.

At present, there exist no screening or treatment guidelines for GaCs with BM (8), and managing BM from GaC can be challenging. Multidisciplinary treatment may enhance long-term survival and QoL (53–55). We found that 7% of GaC cases with BM underwent resection, which may increase the overall survival time for this specific patient population (41, 56, 57). Of note, although surgical resection was correlated with better prognosis in GaC cases with BM, causality and definitive management benefits cannot be derived from this observational investigation. Tailored and timely management is vital, and notably, nowadays, radiotherapy and immune checkpoint inhibitor therapy may be particularly effective for cases with GaC affecting the brain (58). We found that within GaC patients with BM receiving radiotherapy, younger age, antrum/pylorus location, and LN metastasis were correlated with worse survival, which should call for clinicians’ attention.

Gamma-knife stereotactic radiosurgery seems a safe and effective treatment modality for the treatment of BM from GaC with good local control and few side effects, and dose-escalated approaches may improve local control rates (59–61). Radiosurgery appears a nice alternative to surgical resection for cases with BM from advanced gastric cancer (62); gamma-knife radiosurgery and recursive partitioning analysis class 2 may contribute to more favorable clinical outcomes (63). Resection and stereotactic radiosurgery for BM contributed to long-term survival in two Japanese people with BM identified more than 1 year after the removal of primary GaC, who remained alive after 6.5 years and died of non-GaC causes 4 years after surgery, respectively (64). Stereotactic radiotherapy is also associated with better outcomes and longer overall survival after brain relapse (65). Cumulative intracranial tumor volume is a vital prognostic factor in cases with stereotactic radiosurgery-managed gastrointestinal tract cancer BM (66). A case report described a patient with advanced gastric cancer and multiple brain and abdominal LN involvements who was successfully treated through an abscopal effect, which refers to the phenomenon where local radiation therapy is correlated with the regression of metastatic tumor, which is located distantly from the irradiated site, through brain radiation and anti-PD-1 therapies (67). Combined therapy with nivolumab and gamma-knife radiosurgery appeared effective for treating another 55-year-old male with gastric cancer and BM (68). A 68-year-old man with gastric cancer and BM was also reported to be successfully treated with nivolumab (69). Another 63-year-old man with gastroesophageal junction cancer and brain, bone, and gastric intramural metastases was reported to be responsive to combined modality therapy (70). Notably, acquired mutation of *CTNNB1* may drive immune checkpoint inhibitor (ICI)-acquired resistance in microsatellite instability (MSI)-high esophagogastric adenocarcinoma with BM (71).

Agents targeting HER2, pSTAT3, EGFR, and angiogenesis-associated molecules may be feasible for the treatment of BM from gastric cancer (72–78). The combination of apatinib and continual nutritional support may be another option for the treatment of BM from gastric cancer, which may prolong survival (27). HER2+ patients with gastroesophageal adenocarcinoma may have an increased risk to develop brain metastasis (15), and patients with HER2+ gastric cancer may have a higher susceptibility to disease recurrence in the central nervous system (79). The proportion of HER2+ status in patients with gastroesophageal adenocarcinoma and brain involvement appeared higher than expected (80). In cases with gastrointestinal cancer and brain metastases, HER2+ status was more frequent intracranially compared to prior disease sites, suggesting that examining HER2 in brain metastases of those patients might offer additional treatment options, irrespective of HER2 status in previously examined tissues (81). Trastuzumab deruxtecan showed efficacy for the management of a 65-year-old man with BM originating from advanced HER2-positive gastric cancer (82). It would be desirable to investigate if other HER2-targeting antibody-drug conjugates (ADCs; e.g., GQ1001 and T-DM1 (7)) could show similar efficacies against BM from GaCs. BM from advanced gastric cancer was correlated with VEGF expression, and metformin therapy might be helpful for modulating the metastasis capacity *via* reducing the expression of VEGF and blocking epithelial-to-mesenchymal transition (EMT) (83). Arterial infusion chemotherapy with tegafur, epirubicin, and lobaplatin appeared effective for the management of a 73-year-old man with advanced gastric cancer affecting the brain (84). Irinotecan plus cisplatin and irradiation appeared effective for the management of another two Japanese cases with BM from gastric cancer (85). Paclitaxel may also be effective for GaC with BM (86, 87). Accordingly, we found that cases receiving chemotherapy had lower death rates.

The treatment scheme for this specific patient subpopulation should be individualized and based on expected survival, performance status, symptoms, the number, location, and size of metastases, the status of the primary tumor, and dissemination to other organs (61, 88–91). QoL should also be factored into management decisions, especially in the setting of aggressive treatment (17). Searching for ideal treatment pathways for this specific patient subgroup would be greatly desired.

This observational investigation shared some common limitations with other registry-based population-based studies. Information on some other variables that were potentially correlated with BM (e.g., genetic and environmental risk factors) and prognostic variables [e.g., health conditions and comorbidities (92–94)] were not available. HER2 status was not available in the analyzed database. Nonetheless, the majority of the common prognostic and risk factors had been included in multivariable-adjusted models. A few variables (e.g., cancer differentiation grade, local invasion, and LN metastasis) had missing data; they were not initially included in the main multivariable-adjusted analyses but were separately added into the main multivariable-adjusted models to assess the associations with them. Nonsurgical therapies were under-ascertained and registered with low sensitivity but high specificity (35), and detailed data on management [e.g., agents and courses (95)] were not available. Accordingly, they were not included in the main multivariable-adjusted survival analyses, and subgroup analyses for cases having chemotherapy or radiation therapy were specifically done. Furthermore, the findings were based on the US cases and might not be well generalizable to patients from other countries, particularly Asians, in which gastric cancer would be much more prevalent. We would strongly encourage analyses of databases from other nations.

To our knowledge, this report appears to be the largest real-world population-based investigation utilizing robust statistics and patient-level data to address BM in GaC patients. The strict and careful case selection, utilization of comprehensive multivariable-adjusted CR analysis methods, and meticulous stratified investigations enable this study to offer useful, valid, and robust references for personalized management of cases with GaC and BM.

## Conclusions

Among patients with GaC, brain metastasis was correlated with several clinical and pathological factors, which included ethnicity, age, cancer histology, location, LN involvement, and metastases to other sites. Cases with brain involvement had poor survival, which was correlated with age, cancer location, LN metastasis, and management. The findings offer clinically relevant and helpful clues and evidence for individualized treatment of cases with GaC and brain metastasis and future mechanistic explorations.

## Data availability statement

Publicly available datasets were analyzed in this study after signing the appropriate agreement and obtaining the data use approval. This data can be found here: Surveillance, Epidemiology, and End Results Program Research Data (www.seer.cancer.gov).

## Ethics statement

Ethical review and approval was not required for this study using the Surveillance, Epidemiology, and End Results Program database after signing the appropriate agreement and obtaining the data use approval in accordance with the local legislation and institutional requirements. New written informed consent was not required for this study in accordance with the national legislation and the institutional requirements.

## Author contributions

Conception or design: LH, WH, and JZ. Acquisition, analysis, or interpretation of data: LH, LW, YS, YZ, CX, WH, and JZ. Drafting of the manuscript: LH. Critical revision of the manuscript for important intellectual content: LW, YS, YZ, CX, WH, and JZ. Statistical analysis: LH. Administrative, technical, or material support: WH and JZ. All authors have given final approval of the manuscript for submission and publication.

## Funding

This study was supported by the Fund for Medical Center on Aging (GB202103), and the Start-up Fund for the Introduction of High-Level Talents by Ruijin Hospital, Shanghai Jiao Tong University School of Medicine. The funders had no involvement in the study design; in the collection, analysis, or interpretation of data; in the writing of the report; or in the decision to submit the paper for publication.

## Acknowledgments

We are very grateful to the staff of the Surveillance, Epidemiology, and End Results Program for their great work in data collection and delivery and to the members of the Chinese Anti-Cancer Association (CACA), Chinese Society of Clinical Oncology (CSCO), and Chinese Medical Association for all their great support.

## Conflict of interest

The authors declare that the research was conducted in the absence of any commercial or financial relationships that could be construed as a potential conflict of interest.

## Publisher’s note

All claims expressed in this article are solely those of the authors and do not necessarily represent those of their affiliated organizations, or those of the publisher, the editors and the reviewers. Any product that may be evaluated in this article, or claim that may be made by its manufacturer, is not guaranteed or endorsed by the publisher.
